# Supplementary material for: Feed management practices used for dairy cows in confined dairies in Brazil
Source: PLoS One. 2026 Mar 20;21(3):e0344588. doi: 10.1371/journal.pone.0344588 (PMC13004345; doi:10.1371/journal.pone.0344588)
Supplement: S1 Table — (DOCX) [file pone.0344588.s001.docx]

***S1 Table.*** Survey questions with their respective answers’ options.

| Questions | Answer option |
| --- | --- |
| *General farm and herd information* | *-* |
| What state is the farm localized in? | PR^1^, RS^2^, SC^3^, ES^4^, MG^5^, RJ^6^, or SP^7^ |
| What is the main breed used? | Holstein, Holstein x Gyr crosses, Jersey, other |
| What was the average number of lactating dairy cows in the past year? | Open question |
| What systems are your high-production dairy cows housed in? | Free-stall, compost barn, dry lot, and tie-stall |
| Do you receive a nutritionist visit? | Yes/No |
| How often does the nutritionist visit your dairy? | ≤ Weekly, bi-weekly, monthly, >monthly |
| What was the 305-d milk production of your cows in the last year? | ≤6, 6 < to ≤ 7, 7 < to ≤ 8, 8 < to ≤ 9, < 9 to ≤ 10 , < 10 to ≤ 11, 11 < to ≤ 12, < 12 to ≤ 13, or > 13 × 1,000L |
| *TMR preparation and feedstuff evaluation:* | *-* |
| Do you use a wagon mixer? | Yes/No |
| What is the mixing type? | Vertical/horizontal |
| What order are the ingredients loaded in the wagon? | Open question |
| What is the mixing time (min.) after loading the last ingredient? | ≤5, 5 < to ≤ 10,10 < to ≤ 15, < 15 to ≤ 20 |
| Do you check TMR dry matter? | Yes/No |
| What is the TMR dry matter target (%)? | <50, 50 ≤ to ≤ 55, > 55 |
| Do you evaluate TMR particle using a PSPS? How often? | Yes/No; Weekly, biweekly, monthly, > monthly |
| Do you calibrate the mixer wagon scale? How often? | Yes/No; Open |
| Do you use any TMR stabilizers? (e.g. organic acids) | Yes/No |
| Do you send feedstuffs samples for laboratory analysis? (yes/no) How often? | Yes/No; Open |
| Do you evaluate TMR physically effective NDF? (yes/no) How often? | Yes/No; Open |
| Do you evaluate corn silage particle size at harvest? (yes/no) How often? | Yes/No; Open |
| *Feed bunk practices*: | *-* |
| How many times a day is the TMR fed? | 1, 2, 3, 4 |
| Do you do feed push-ups? How many times a day? | Yes/No; 1, 2, 3 4, ≥ 5 |
| Do you clean up the feed bunk? | Yes/No |
| Do you feed for refusals? What percentage? | Yes/No; Open |
| Do you measure feed efficiency? How often? | Yes/No; Weekly, bi-weekly, monthly |
| *High production cows management*: | *-* |
| What is the feed bunk space? | Open question |
| What is the actual pen stocking density? | Open question |
| Do you feed the high-production cows group immediately after milk time? | Yes/No |
| Do you group primiparous separated from multiparous cows? | Yes/No |
| Do you have a water trough wash protocol? | Yes/No |
| Do you have a cooling system? | Yes/No |
